# Impact of Chronic Smoking on Meibomian Gland Dysfunction

**DOI:** 10.1371/journal.pone.0168763

**Published:** 2016-12-28

**Authors:** Shen Wang, Hui Zhao, Caihong Huang, Zhengri Li, Wei Li, Xiaobo Zhang, Zuguo Liu

**Affiliations:** Eye Institute of Xiamen University, Xiamen University Medical College, Xiamen, Fujian, China; Ocular Surface Center, UNITED STATES

## Abstract

**Purpose:**

The aim of this study was to explore the relationship between chronic cigarette smoking and meibomian gland dysfunction (MGD).

**Methods:**

This study enrolled 322 smokers with MGD and 2067 non-smokers with MGD. All enrolled subjects were tested in the following sequence: Ocular Surface Disease Index (OSDI), tear film breakup time (TBUT), corneal fluorescein staining (CFS), Schirmer I test (SIT)and finally slit-lamp microscope examination of lid margin abnormalities, meibomian gland expression as well as meibum.

**Results:**

Compared with the MGD patients without smoking, the MGD patients with smoking had significantly increased scores of lid margin abnormality and meibum (P<0.01 for each comparison). No significant difference was noted in OSDI, TBUT, CFS, SIT or the score of Meibomian gland expressibility between the smokers and non-smokers (P>0.05 for each comparison). In the smokers, the smoking index was significantly correlated with the scores of lid margin abnormality (Both sexes, R = 0.19, P<0.01; Male, R = 0.18, P<0.01) and meibum (Both sexes, R = 0.29, P<0.01; Male, R = 0.20, P<0.01), whereas it was not significantly correlated with OSDI, TBUT, CFS, SIT or score of Meibomian gland expressibility (P>0.05 for each comparison).

**Conclusion:**

The findings of this study suggest chronic smoking might be associated with MGD.

## Introduction

Tobacco smoking is one of the most prevalent addictive habits, and has deteriorating effects on numerous diseases, such as cardiovascular, respiratory and malignant diseases[1–3]. Smoking also has adverse ocular effects. It has been well documented that cigarette smoking is associated with many ophthalmological disorders including glaucoma, optic neuritis, diabetic retinopathy, age-related macular degeneration and ocular inflammation [4–6]. The recognition about the relationship between smoking and ophthalmologic abnormalities continues to progress.

Meibomian gland dysfunction (MGD) is an ocular surface disease with chronic and diffuse abnormality of the meibomian glands. It is characterized by terminal duct obstruction and/or qualitative/quantitative changes in the glandular secretion[7]. The prevalence of MGD reported in literature varies widely, from 3.5% to almost 70%[8].Population-based studies have reported that the prevalence of MGD in Asia is higher than that in other populations. It has been reported the prevalence of MGD is 46.2% in Thailand[9],60.8% in Taiwan[10], 61.9% in Japan[11], and 69.3% in China[12]. MGD has a tremendous negative impact on activities of daily life. It may result in alteration of the tear film stability, symptoms of eye irritation and clinically apparent inflammation in ocular surface [8]. Until now, little has been reported regarding the impact of tobacco smoking on MGD disease.

The purpose of this study was to explore the relationship between chronic cigarette smoking and MGD.

## Materials and Methods

### Subjects

This study enrolled a total of 2389 patients with MGD (322 smokers, 40 female and 282 male, aged45.5±0.78 years; 2067 non-smokers, 1552 female and 515 male, aged44.7±0.40 years) from July, 2014 to Jan, 2015.No significant difference was found in age between the two groups(P>0.05). Significant difference was found in sex between the two groups(P<0.01). MGD was diagnosed by the following evidence as previously reported[13]: 1) presence of ocular symptoms; 2) at least one lid margin abnormality including irregular lid margin, vascular engorgement, plugged meibomian gland orifices and anterior or posterior displacement of the mucocutaneous junction and 3) impaired meibum expression. Ocular symptom was determined by Ocular Surface Disease Index (OSDI), and the subject with score of > 12 was considered abnormal[14]. This study included the smokers who consumed at least 10 cigarettes per day for at least the prior 5 years. The smoking index was calculated as follows: Smoking index = (number of cigarettes consumed per day)×(years of smoking). None of the control subjects had actively smoked cigarettes or had a history of passive smoking exposure at home or at work. Potential participant with any of the following conditions was excluded from the study: Sjögren’s syndrome, Stevens Johnson syndrome, past ocular surgery, history of ocular trauma or chemical burn, infectious keratoconjunctivitis, contact lens wearing or other ocular diseases.

Clinical data of this study was collected from eight clinical centers, including The Second Hospital of Jilin University, Tongji Hospital of Tongji Medical College, Peking University People’s Hospital, Shanghai General Hospital, The First Affiliated Hospital of Guangxi Medical University, The Fourth Affiliated Hospital of China Medical University, West China Hospital of Sichuan University and Fujian Medical University Union Hospital. The study procedures in this study adhered to the Declaration of Helsinki that guides studies involving human subjects. This study was approved by the institutional review board of Xiamen University, and written consent was obtained from all of the participants.

### Experimental Procedure

All enrolled subjects were tested in the following sequence: OSDI, tear film breakup time (TBUT), corneal fluorescein staining (CFS), Schirmer I test without anesthetic (SIT)and finally slit-lamp microscope examination of lid margin abnormalities, meibomian gland expression as well as meibum. All the measurements were performed for one eye of each subject randomly.

### Tear Film Breakup Time

After instillation of 1 μL of 1% fluorescein preservative-free solution in the conjunctival sac with a micropipette, the patients were instructed to blink several times for a few seconds to ensure adequate mixing of the dye. The interval between the last complete blink and appearance of the first corneal black spot in the stained tear film was measured 3 times, and the mean value of the measurements was calculated.

### Corneal Fluorescein Staining

CFS was graded as previously reported[15]from 0 to 12, a sum of the scores of corneal four quadrants, which were scored individually as 0 (no staining), 1 (mild staining with a few scattered dots of stains), 2 (moderate staining between 1 and 3), and 3 (severe staining with confluent stains or corneal filaments).

### Schirmer І Test

SIT without topical anesthesia was performed to measure the tear production. A 5×35-mmfilter paper strip was inserted in the lateral one third of the inferior fornix. After 5 minutes, the length that had become wet by the tear film was measured. Readings were recorded in millimeters (mm).

### Lid Margin Abnormality

As described in detail previously[16–18],four lid margin abnormalities (irregular lid margin, vascular engorgement, plugging of meibomian gland orifices and anterior or posterior replacement of the mucocutaneous junction) were scored from 0 through 4 according to the number of these abnormalities present in each eye.

### Meibomian Gland Expressibility

As described in detail previously[18], meibomian gland expressibility from the central 8 meibomian glands on the upper eyelid was graded on a 4-point scale as previously reported (0 = all glands expressible, 1 = 3–4glands expressible, 2 = 1–2 glands expressible, 3 = no glands expressible) following firm digital pressure to the eyelid margins.

### Meibum

As described in detail previously[18], meibum score from the central 8 meibomian glands on the lower and upper eyelid was graded on a 4-point scale (0 = clear fluid, 1 = cloudy fluid, 2 = cloudy particulate fluid, 3 = inspissated like toothpaste) following firm digital pressure to the eyelid margins.

### Data Analysis

Data were presented as mean ± standard error (SE) for all the variables. Analysis of covariance test controlling for sex was performed to determine the differences of each variable between the smokers and non-smokers of both sexes. Analysis of variance was performed to determine the differences of each variable between the male smokers and non-smokers. Partial correlation controlling for sex was used to analysis the relation between smoking index and other variables in smokers of both sexes. Pearson correlation was used to analysis the relation between smoking index and other variables in male smokers. P-values less than 0.05 were considered significant. SPSS software (SPSS version 13.0, Inc., Chicago, Illinois, USA) was used to analyze all data in this study.

## Results

### The Effect of Smoking on MGD Parameters

As shown in Table 1, in the MGD patients, compared with the non-smokers, the smokers had significantly increased scores of lid margin abnormality and meibum (P<0.01 for each comparison). No significant difference was noted in OSDI, TBUT, CFS, ST-I or the score of meibomian gland expressibility between the smokers and non-smokers in this study (P>0.05 for each comparison).

**Table 1:** The Effect of Smoking onmeibomian gland dysfunction (MGD) Parameters.

Parameters	Smokers	Non-smokers
**Both sexes**		
OSDI	36.15±0.93	35.85±0.39
TBUT	4.00±0.14	3.96±0.05
CFS	1.70±0.15	1.84±0.06
SIT	6.29±0.30	6.92±0.15
Lid margin abnormality score**	0.78±0.04	0.60±0.02
Meibomian gland expressibility score	1.26±0.05	1.37±0.02
Meibum Score**	1.03±0.05	0.82±0.02
**Male**		
OSDI	35.77±0.98	34.62±0.70
TBUT	4.10±0.16	4.05±0.11
CFS	1.57±0.15	1.78±0.11
SIT	6.43±0.32	7.15±0.28
Lid margin abnormality score**	0.80±0.04	0.62±0.03
Meibomian gland expressibility score	1.21±0.06	1.32±0.04
Meibum Score**	1.05±0.06	0.81±0.04

Data shown as mean ± SE;

** P<0.01

OSDI: Ocular Surface Disease Index; TBUT: tear film breakup time; CFS: corneal fluorescein staining; SIT: Schirmer I test.

### The Correlation between Smoking Index and MGD Parameters in the Smokers

**Fig 1:**
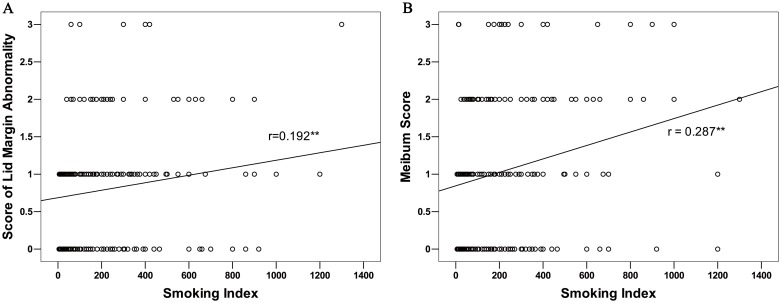
The Correlation between Smoking Index and MGD Parameters in the Smokers of both sexes. In the smokers with meibomian gland dysfunction (MGD), the smoking index was significantly correlated with the scores of lid margin abnormality (A) and meibum (B).

**Fig 2:**
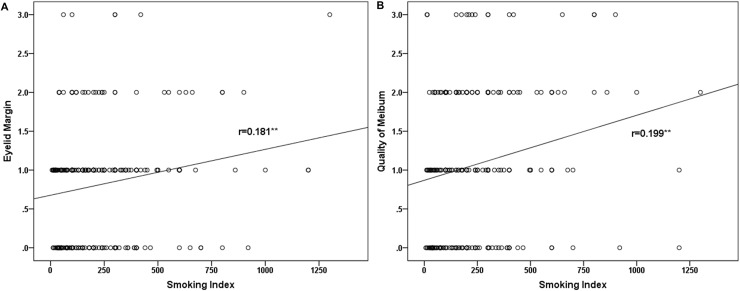
The Correlation between Smoking Index and MGD Parameters in the Male Smokers. In the male smokers with meibomian gland dysfunction (MGD), the smoking index was significantly correlated with the scores of lid margin abnormality (A) and meibum (B).

In the smokers, the smoking index was significantly and positively correlated with the scores of lid margin abnormality (Both sexes, R = 0.19, P<0.01, Fig 1A; Male, R = 0.18, P<0.01, Fig 2A) and meibum (Both sexes, R = 0.29, P<0.01, Fig 1B; Male, R = 0.20, P<0.01, Fig 2B), whereas it not significantly correlated with OSDI, TBUT, CFS, ST-I or the score of meibomian gland expressibility in the MGD patients (P>0.05 for each comparison).

## Discussion

MGD is a prevalent problem with severe detriments to well-being. It is necessary that studies be undertaken to explore associated risk factors of MGD. Until now, the risk factors for MGD remain largely unknown. Cigarette smoking is a tremendous source of toxic substances intake, and has many adverse effects on different organs, including the eye[2,4–6].To the best of our knowledge, little has been reported about the relationship between smoking and MGD. Viso et al.[19] reported in 2012 that past smoking was a risk factor for MGD, whereas current smoking was not. The current study demonstrated for the first time that current cigarette smoking might be associated with MGD. This study found that the scores of lid margin abnormality and meibum in smokers were significantly lower than those in non-smokers in the MGD patient. In addition, the smoking index was significantly and positively correlated with the scores of lid margin abnormality and meibum in smokers with MGD.

A growing body of evidence suggests that the core mechanism of MGD is an obstructive process that is caused by hyperkeratinization of the meibomian duct and increased viscosity of meibum[7]. In addition, the pathogenesis of MGD is also interact with some associated functional complexes such as an aberrant differentiation of stem cells, an increased growth of bacteria and increased inflammatory mediators inside the meibomian glands and/or on the lid margin[7]. Although the exact mechanism by which tobacco smoking has an influence on MGD remains unknown, and perhaps more than one mechanism are involved, we speculate that the increased inflammatory reactions evoked by smoking may involved in the association between smoking and MGD. It has been suggested that smoking may cause inflammatory reactions and disturbances in the immune system. Chronic cigarette smoking may affect both the innate and adoptive immune response. Smokers have a decreased T-suppressor lymphocyte activity and a lower level of immunosuppression than non-smokers[20,21]. It has also been confirmed cigarette smoking may augment the production of numerous pro-inflammatory cytokines such as tumor necrosis factor (TNF)-α, interleukin (IL)-1, IL-6, IL-8 and decrease the levels of anti-inflammatory cytokines such as IL-10[22]. Further studies are needed to determine the effect of cigarette smoking on the meibomian gland on a molecular basis in terms of inflammation.

Although ocular irritation caused by active or passive cigarette smoking has been reported[5,6], the impact of chronic smoking on ocular discomfort caused by MGD has not been studied in detail. OSDI is a psychometrically tested, valid, and reliable instrument for measuring the severity of ocular discomfort in ocular surface disease such as dry eye[23]. This study used OSDI to evaluate the relationship between chronic smoking and ocular discomfort in MGD patients, and found that no significant difference was noted in OSDI between smokers and non-smokers, suggesting chronic smoking may not exacerbate ocular discomfort caused by MGD. The anesthetic effect of tobacco smoking on the ocular surface may explain the lack of association observed between ocular discomfort and current smoking in MGD patient. It has been confirmed that, in dry eye patients, the current smokers had a significant decreased corneal and conjunctival sensitivity compared to non-smokers[24].

It has been suggested that the ocular surface (cornea, conjunctiva, accessory lacrimal glands and meibomian glands), the main lacrimal gland and the interconnecting innervations interact with each other to function as a whole unit[7,25–27]. If any portion of this functional unit is damaged, the whole unit which maintains the normal physiological function of tear film and ocular surface will be impeded. Therefore, theoretically, the lid margin and meibum abnormality exacerbated by chronic smoking may led to decreased tear production, decreased tear stability and increased ocular surface damage. Indeed, although the effect of smoking on tear system and ocular surface remain on debate, evidences have suggested that chronic smoking or passive smoking may imply the tear system and cause ocular surface epithelium damage[5,6,24,28]. Incredibly, no significant difference was noted in TBUT, CFS or SIT between smokers and non-smokers in the present study. This result is possibly due to the use of invasive method to measure the tear characteristics in the present study. The use of fluorescein and filter paper strip might cause reflex tearing and alter the tear system, resulting in great variation in these tests. It is necessary to further confirm the effect of chronic smoking on tear system using minimal invasive tear parameters such as non-invasive BUT and phenol red cotton test in multicenter large scale studies. Previously, it has been reported that decreased epithelial density evaluated by corneal confocal microscopy in vivo may appear before disruption of epithelial barrier function evaluated by CFS, and may be more sensitive to reflect the epithelial damage than fluorescein staining in dry eye[29].Further studies are also needed to explore the effect of chronic smoking on corneal epithelial damage at on a cellular level using corneal confocal microscopy.

In summary, the findings of this study suggest chronic smoking might be associated with MGD.
